# Endoscopic features of colorectal lymphoma according to histological type

**DOI:** 10.1002/jgh3.12738

**Published:** 2022-04-06

**Authors:** Tatsuo Yachida, Takahisa Matsuda, Taku Sakamoto, Takeshi Nakajima, Yasuo Kakugawa, Akiko Miyagi Maeshima, Hirokazu Taniguchi, Ryoji Kushima, Kensei Tobinai, Hideki Kobara, Hisashi Masugata, Tsutomu Masaki, Yutaka Saito

**Affiliations:** ^1^ Endoscopy Division National Cancer Center Hospital Chuo City Tokyo Japan; ^2^ Department of Gastroenterology and Neurology, Faculty of Medicine Kagawa University Kita‐gun Kagawa Japan; ^3^ Department of General Internal Medicine, Faculty of Medicine Kagawa University Kita‐gun Kagawa Japan; ^4^ Department of Pathology National Cancer Center Hospital Chuo City Tokyo Japan; ^5^ Pathology and Clinical Laboratory Division JR Tokyo General Hospital Shibuya City Tokyo Japan; ^6^ Department of Clinical Laboratory Medicine and Diagnostic Pathology Shiga University of Medical Science Otsu Shiga Japan; ^7^ Department of Hematology National Cancer Center Hospital Chuo City Tokyo Japan

**Keywords:** colon, colonoscopy, lymphoma

## Abstract

**Background and Aim:**

This study aimed to investigate the relationship between the histological type of colorectal lymphoma and its endoscopic features.

**Methods:**

We retrospectively analyzed patients with primary colorectal lymphoma who were diagnosed using colonoscopy and biopsy specimens at the National Cancer Center Hospital, Tokyo, Japan. The lesions were macroscopically classified into the following types via colonoscopy: polypoid, ulcerative, multiple lymphomatous polyposis, diffuse, and mixed.

**Results:**

A total of 117 lesions were identified in 90 patients enrolled in this study. Of these, 59 (50%) were located in the ileocecal region, 23 (20%) in the rectum, 9 (8%) in the transverse colon, 8 (7%) in the sigmoid colon, 7 (6%) in the descending colon, and 4 (3%) in the ascending colon. Moreover, the most common histological subtypes were diffuse large B‐cell lymphoma (DLBCL) in 39 patients (43%) and mantle cell lymphoma (MCL) in 23 patients (26%), followed by follicular lymphoma (FL; 17%), mucosa‐associated lymphoid tissue (MALT) lymphoma (9%), peripheral T‐cell lymphoma‐NOS (2%), monomorphic epitheliotropic intestinal T‐cell lymphoma (MEITL; 2%), and Burkitt lymphoma (1%). More than half of the DLBCL (52%), MCL (52%), and MALT (56%) lymphomas were macroscopically classified as polypoid types. In contrast, FL lesions showed various macroscopic types. The majority of DLBCL (62%) and FL (78%) lesions were distributed in the ileocecal region. MCL lesions tended to be widely spread in various sites of the large intestine.

**Conclusions:**

Colorectal lymphomas showed macroscopically distinctive features depending on the histological type. Understanding the macroscopic classification of the lesions by colonoscopy and its distribution may be helpful in diagnosing the type of lymphoma and determining the malignant grade based on the histological types.

## Graphical abstract

This study aimed to investigate the relationship between the histological type of colorectal lymphoma and its endoscopic features. Colorectal lymphomas showed macroscopically distinctive features depending on the histological type.

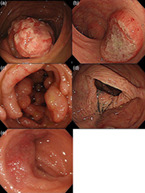

## Introduction

Approximately 40% of lymphomas are extranodal, and the most common site of extranodal involvement is the gastrointestinal (GI) tract^1^; however, occurrence of colorectal lymphoma is rare in the GI tract. Primary lymphomas arise less frequently from the large intestine than either gastric or small bowel lymphomas. Colorectal lymphomas account for only 10–20% of GI lymphomas, a lower rate of involvement than either stomach (50–60%) or small intestine (20–30%) involvement.^2^, ^3^, ^4^, ^5^ Primary lymphoma of the colorectum is exceedingly rare and comprises 0.2–1.2% of all colorectal tumors.^3^, ^6^, ^7^, ^8^ In the 1960s, Dawson's criteria (1961), which are limited to cases with a stage I/II, were widely used; however, in recent years, the Lewin criteria (1978) has been applied. If the lesion is located mainly in the GI tract, this is considered to be the primary organ regardless of the stage of the disease.^9^, ^10^ In fact, in advanced stages of GI lymphoma, differentiating primary lymphoma of the GI tract from GI invasion of systemic lymphoma is often difficult. Therefore, the method for differentiating primary colorectal lymphoma from systemic lymphoma involving the colorectum has not yet been fully established.

The most common subtype of lymphoma in the colorectum is non‐Hodgkin's lymphoma (NHL).^2^ In the previous systemic review of 1524 patients across 23 studies, Lightner *et al*. demonstrated that primary GI NHL occurred most frequently in the ileocecum (37.2%) and were most commonly classified as diffuse large B‐cell lymphoma (DLBCL) (53.6%).^11^ Previous studies have reported that the main examination required for morphological diagnosis was X‐ray examination; however, in recent years, patients with colorectal lymphomas have undergone colonoscopy at our institution for diagnosis. Several macroscopic lymphoma types have been confirmed with detailed examination; however, the endoscopic features remain unknown due to their rarity.

This study aimed to investigate the relationship between the histological type of colorectal lymphoma and its endoscopic features.

## Methods

We retrospectively analyzed all 90 patients with primary colorectal lymphoma who were diagnosed using colonoscopy and biopsy specimens between January 2000 and October 2011 at the National Cancer Center Hospital, Tokyo, Japan. Written informed consent for endoscopic examination and general consent for researches were obtained from all patients before the procedures. All cases satisfied the criteria for primary GI lymphoma as defined by Lewin *et al*.^10^ A method for discriminating primary colorectal lymphomas from systemic lymphomas involving the large intestine has not been fully developed, but in this study we considered a case to be colorectal primary lymphoma if the main bulk of the lesions was located in the colorectum according to previous reports.^12^, ^13^, ^14^ All histological materials were obtained by endoscopic biopsy. Then, these tissue specimens were stained routinely with hematoxylin and eosin (H&E). However, pathologically, differential diagnosis can be extremely difficult because of similar cytologic features and common presence of lymphoepithelial lesions. Immunohistochemical detection of characteristic markers is the best way of differentiation. Therefore, in addition, immunohistochemical analysis was performed using antibodies against the following antigens for diagnosis: CD3, CD5, CD10, CD20, CD56, BCL2, BCL6, cyclin D1, MUM1, cMYC, and Ki67. All specimens were diagnosed by two hematopathologists according to the World Health Organization (WHO) classification.^15^

**Figure 1:**
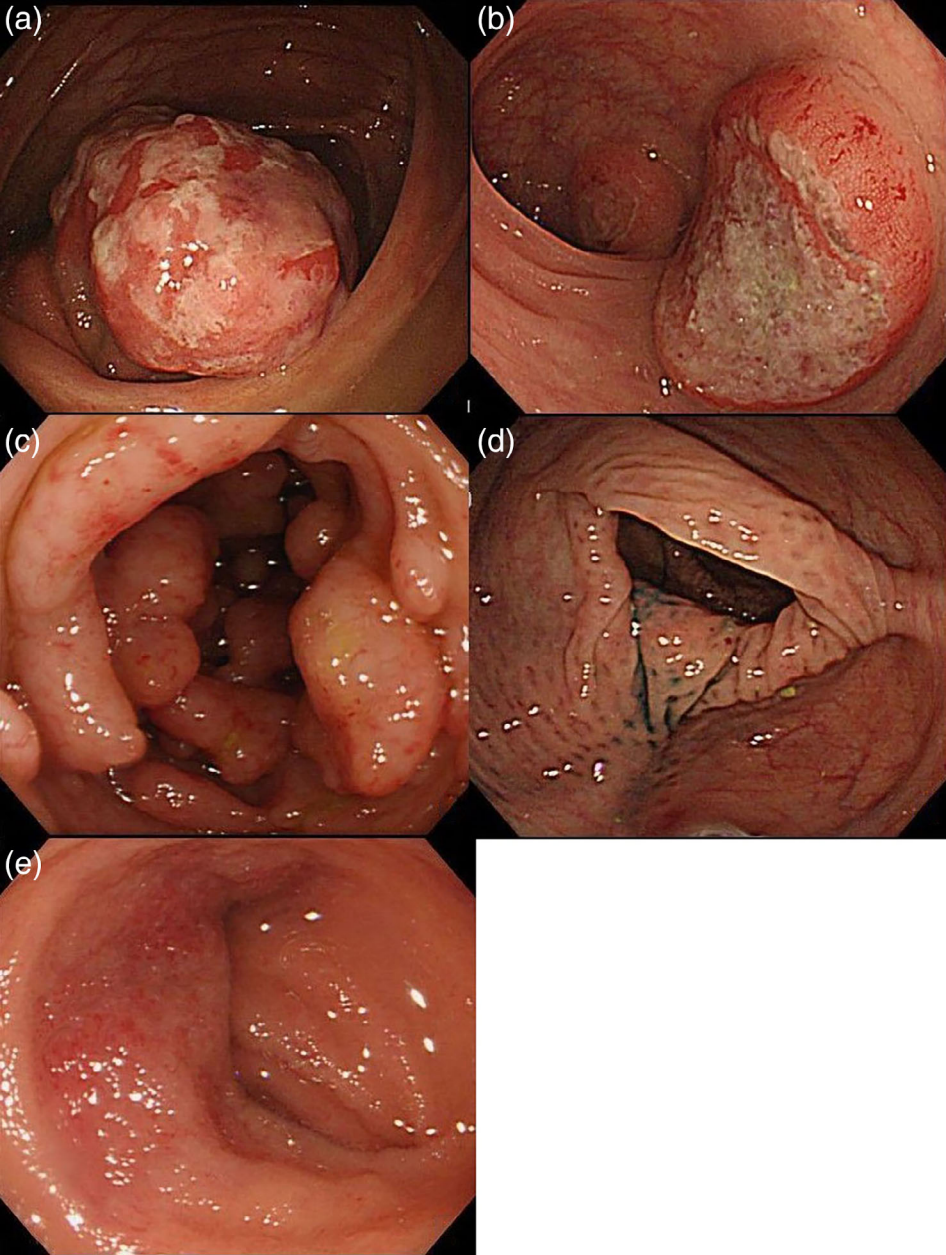
(a) Polypoid type, diffuse large B‐cell lymphoma (DLBCL) in the cecum. (b) Ulcerative type DLBCL transformed from follicular (FL) in the rectum. (c) Multiple lymphomatous polyposis, FL in the terminal ileum. (d) Diffuse type, mucosa‐associated lymphoid tissue in the whole colorectum. (e) Mixed types/other type, mantle cell lymphoma in the terminal ileum.

The clinicopathological variables recorded from the clinical, endoscopic, and pathological records were age, gender, endoscopic findings (lesion location and macroscopic type), and histopathologic diagnosis. According to previous reports, all endoscopic findings were diagnosed by reviewing the images, and the macroscopic type was classified as follows: (i) polypoid type (solitary or fewer than 10 elevated lesions forming tumorous nodules; these lesions often resemble submucosal tumors and sometimes accompany ulcers on their tops); (ii) ulcerative type (solitary or multiple lowered lesions due to ulcers); (iii) multiple lymphomatous polyposis (MLP) type (multiple micropolyps with or without some large polyps; 10 or more polyps); (iv) diffuse type (diffuse changes in the mucosal color and/or changes in mucosal morphology); and (v) mixed type (combinations of these four subtypes, Fig. 1a–e).^16^, ^17^

## Results

### Patient demographic details

A total of 117 lesions in 90 patients were identified in this study. The median age was 64 (range: 29–84) years, and 58 (64%) were men. In 17 patients (19%), the lesions were located only in the colon or rectum. In the remaining cases, the lesions were present simultaneously in the colon/rectum and in other organs (including lymph nodes).

### Lymphoma distributions and macroscopic types

In this study, out of 117 lesions, 59 (50%) lesions were located in the ileocecal region, 23 (20%) in the rectum, 9 (8%) in the transverse colon, 8 (7%) in the sigmoid colon, 7 (6%) in the descending colon, and 4 (3%) in the ascending colon. Moreover, 26 (29%) patients had synchronous lesions that were located in two or more sites and 7 (8%) patients had one lesion spreading throughout the colorectum (Tables 1 and 2).

**Table 1:** Distribution of the lesion sites

Distribution (%)	*n* = 117^†^
Ileocecal region	59 (50%)
Ascending colon	4 (3%)
Transverse colon	9 (8%)
Descending colon	7 (6%)
Sigmoid colon	8 (7%)
Rectum	23 (20%)
Whole colorectum	7 (6%)

^†^ Twenty‐six (29%) of 90 patients had synchronous tumors that were located in two or more sites.

**Table 2:** Macroscopic types of the lesions

Macroscopic type (%)	*n* = 117
Polypoid type	51 (44%)
Ulcerative type	26 (22%)
MLP	14 (12%)
Diffuse type	16 (14%)
Mixed type/other type	10 (8%)

MLP, multiple lymphomatous polyposis.

Of the 117 lesions, 51 (44%) were macroscopically classified as polypoid, 26 (22%) as ulcerative, 14 (12%) as MLP, 16 (14%) as diffuse, and 10 (9%) as mixed/other types. The majority of the macroscopic types were polypoid and ulcerative.

### Histological classification

The primary colorectal lymphomas in 86 (95%) and 4 (5%) patients were of the B‐cell and T‐cell lineage, respectively (Table 3). Of the 90 patients, 39 (43%) were histologically classified with DLBCL, 23 (26%) with mantle cell lymphoma (MCL), 15 (17%) with follicular lymphoma (FL), 8 (9%) with mucosa‐associated lymphoid tissue lymphoma (MALT), 2 (2%) with peripheral T‐cell lymphoma‐NOS, 2 (2%) with monomorphic epitheliotropic intestinal T‐cell lymphoma (MEITL), and 1 (1%) with Burkitt lymphoma. The most frequent histological type was DLBCL.

**Table 3:** Histological classification of lesions

Histological type	*n* = 90
B‐cell lymphoma	
Diffuse large B‐cell lymphoma	39 (43%)
Mantle cell lymphoma	23 (26%)
Follicular lymphoma	15 (17%)
Mucosa‐associated lymphoid tissue lymphoma	8 (9%)
Burkitt like lymphoma	1 (1%)
T‐cell lymphoma	
Peripheral T‐cell lymphoma‐NOS	2 (2%)
Monomorphic epitheliotropic intestinal T‐cell lymphoma	2 (2%)

### Relationship between histological and macroscopic lymphoma types

More than half of the DLBCL (52%), MCL (52%), and MALT (56%) lesions were macroscopically classified as polypoid. By contrast, FL tumors were of various macroscopic types (Table 4).

**Table 4:** Relationship between histological types and macroscopic types of the lesions

	DLBCL (*n* = 48)	MCL (*n* = 33)	FL (*n* = 18)	MALT (*n* = 9)	Other (*n* = 9)
Polypoid type	25 (52%)	17 (52%)	4 (22%)	5 (56%)	0
Ulcerative type	18 (38%)	1 (3%)	1 (6%)	0	6 (67%)
MLP	1 (2%)	6 (18%)	5 (28%)	1 (11%)	1 (11%)
Diffuse type	2 (4%)	5 (15%)	5 (28%)	2 (22%)	2 (22%)
Mixed type/other type	2 (4%)	4 (12%)	3 (16%)	1 (11%)	0

DLBCL, diffuse large B‐cell lymphoma; FL, follicular lymphoma; MALT, mucosa‐associated lymphoid tissue; MCL, mantle cell lymphoma; MLP, multiple lymphomatous polyposis.

### Proportion of histological types according to macroscopic lymphoma types

**Figure 2:**
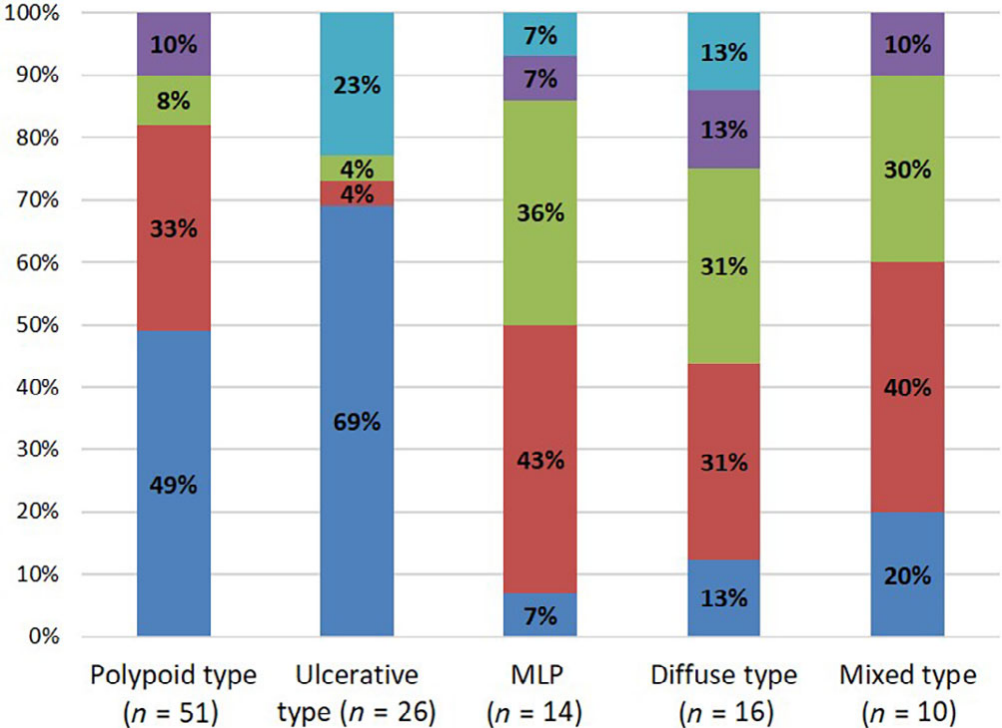
Proportion of histological types according to macroscopic types of the lesions. (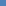), diffuse large B‐cell lymphoma; (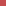), mantle cell lymphoma; (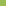), follicular lymphoma; (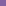), mucosa‐associated lymphoid tissue; (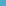), other. MLP, multiple lymphomatous polyposis.

The majority (25/51, 49%) of the polypoid lymphoma was DLBCL, followed by MCL (17/51, 33%). Similarly, the majority (18/26, 69%) of the ulcerative lymphoma was DLBCL (Fig. 2). Most of the MLP lymphoma was histologically classified as MCL (6/14, 43%) and FL (5/14, 36%). Among the diffuse types, MCL and FL were observed with the same frequency (5/16, 31%).

### Relationship between histological types and distributions of the lymphomas

**Figure 3:**
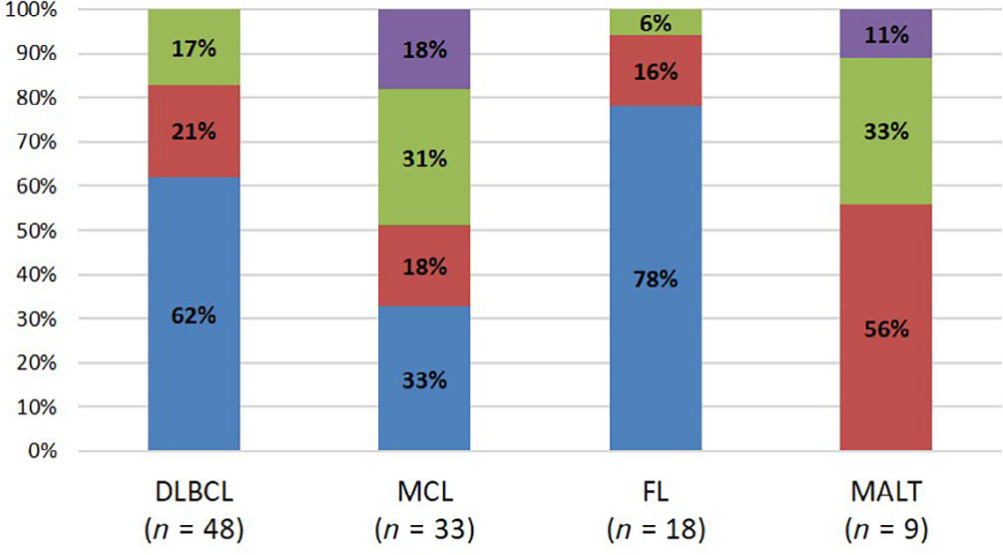
Relationship between histological types and distribution of the lesions. (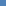), Ileocecal region; (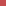), colon (ascending‐sigmoid); (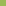), rectum; (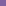), whole colorectum. DLBCL, diffuse large B‐cell lymphoma; FL, follicular lymphoma; MALT, mucosa‐associated lymphoid tissue; MCL, mantle cell lymphoma.

A majority of DLBCL (30/48, 62%) and FL (14/18, 78%) lesions were distributed in the ileocecal region (Fig. 3). MCL lesions tended to be widely spread in various sites of the large intestine. Moreover, 10 (31%) MCL lesions and 3 (33%) MALT lesions were located in the rectum.

## Discussion

This study has a larger number of cases than in previous reports and first revealed that detailed endoscopic features of primary colorectal lymphoma cases differed by their cellular origin.

It is thought that some primary NHLs of the colon arises from mucosa‐associated lymphatic tissue of the colon.^2^ Risk factors for colonic NHL are autoimmune diseases such as inflammatory bowel disease, celiac disease, and human immunodeficiency virus carrier status.^18^ The most common symptoms are abdominal pain, weight loss, abdominal mass, and blood in the stool, as well as nausea, vomiting, altered bowel habits, obstruction, and acute peritonitis due to intussusception and intestinal perforation.^19^ Complete intestinal obstruction is a very rare clinical manifestation because colorectal lymphoma is more malleable than adenocarcinoma and does not promote connective tissue hyperplasia; therefore, incomplete intestinal obstruction is more common. GI lymphoma is staged according to the Lugano classification, which has been adopted by the eighth edition of the Union for International Cancer Control (UICC) TNM classification.^20^ The treatment of colonic lymphoma includes chemotherapy alone or a combination of surgery and chemotherapy depending on the disease stage.

The GI tract is the most common site of extranodal lymphoma occurrence, which affects 6–12% of all patients.^21^, ^22^ The stomach remains the most common site of primary GI lymphoma; however, primary colorectal lymphoma is a rare diagnosis. Generally, in some cases, this represents secondary invasion of the colon or rectum by a widespread systemic lymphoma. In this study, 81% of patients also had tumors in other organs, including secondary infiltration from systemic lymphoma. In addition, various types of lymphomas occur in the GI tract with variable frequencies, either as primary disease or as part of systemic involvement. Primary lymphoma of the GI tract, if strictly defined, refers to an extranodal lymphoma arising in a specific site of the GI tract, with the bulk of disease localized to the site, with or without regional lymph node involvement. However, less stringent inclusion criteria are commonly used, allowing for contiguous involvement of other organs and for distant nodal disease, provided that the extranodal tumor is the presenting site and constitutes the predominant disease. Therefore, results reported in different studies are not necessarily comparable because of the use of different inclusion criteria.^23^

Primary GI lymphomas are usually reported in the fifth to sixth decades of life; however, all age groups were included in the current study.^24^, ^25^ In other studies, the maximum incidence was found in the 50–70‐year age group, with the mean age range of presentation of 50–55 years.^26^, ^27^, ^28^ In our study, the median age was 64 years with similar results. The disease appears later in life, predominantly in the male population.^29^, ^30^, ^31^ Our study showed a male predominance, which correlates well with the results of most of the previous studies. As observed in other studies, histologically, the most common subtype of lymphoma in the colon is NHL^2^; the most common subtype of lymphoma found in our study was DLBCL, which is similar to that reported in previous studies (43–63%).^8^, ^13^, ^28^, ^32^, ^33^ According to the WHO *Classification of Tumors of the Digestive System Tumors*, Fifth edition, the majority of colorectal lymphomas (>50%) are DLBCLs, which includes immunodeficiency‐associated cases, followed by MALT, FL, MCL, and Burkitt lymphomas.^23^, ^34^ The frequency of DLBCL, MCL, and FL was high in the present study; however, the frequency of MALT lymphoma was lower than the frequency reported by the WHO.^23^ The difference between previous reports and our study may be attributed to the fact that our hospital, being a cancer center, consults patients with many different types of malignant lymphoma; also, our study included only Japanese patients. The most common location of colorectal lymphoma is the cecum, occurring in 30–60% of patients,^5^, ^8^, ^9^, ^10^, ^11^, ^14^, ^29^, ^32^, ^35^, ^36^ followed by that of the sigmoid and rectum, which accounts for 10–25% of the colorectal lymphomas. In this study, the most common sites observed were the cecum and rectum. This is presumably due to the excess lymphatic tissue in this region.

Regarding the relationship between the macroscopic and histological type of lymphoma, previous reports have shown that the majority of MLP lymphomas consisted of FL and MCL lesions, which is similar to our findings.^37^, ^38^, ^39^ In a previous study, the most frequent macroscopic type of intestinal lymphoma involving the small intestine was ulcerative, followed by polypoid lymphoma.^17^ Macroscopically, in our study, most lymphomas were polypoid, followed by ulcerative. The depth of invasion of ulcerative lymphomas is more than that of the polypoid type, and the lymphoma's macroscopic type and the depth of invasion may be related. Since most of the tumors were macroscopically classified as polypoid in the present study, it is possible that our study was limited by the observation range of the colonoscopy (colorectum and ileocecal region) except the small intestine. Additionally, colonoscopy was performed during the early stage of the lesions, and it is likely that the lesions were detected before the tumor changed from polypoid to ulcerative. Furthermore, some patients underwent colonoscopy even when they did not have symptoms to diagnose the disease stage while in the hospital.

There are only a few studies on primary colorectal lymphoma. Further studies are needed to check the difference between a primary colorectal lymphoma and a large intestine lesion, a secondary lesion related to systemic illness.

### Limitations

The limitations of this study were that our hospital is a cancer center, which consults patients with different types of malignant lymphomas. Therefore, the histological type and progression of malignant lymphoma may be biased. The data in this study existed only for the period described, so the study is based on older data. In addition, colorectal lymphoma is a disease occurring simultaneously in other organs, whereas this study included cases where it existed in multiple locations in the GI tract. Moreover, in this study, we have not been able to examine the symptoms, staging, treatment, and outcomes in each of our cases, and also considered only Japanese patients.

In summary, colorectal lymphomas showed macroscopically distinctive features depending on the histological type. Although it is true that pathological diagnosis by biopsy is the most reliable diagnostic modality, endoscopic findings can also be a sufficient supplemental predictor. Understanding the macroscopic classification of the lesions by colonoscopy and its distribution may be helpful in diagnosing the type of lymphoma and determining the malignant grade based on the histological types.
